# Personal

**Published:** 1902-04

**Authors:** 


### PERSONAL.

Dr. Robert M. Andrews, U. of B. '98, has been appointed health officer of Bergen in the place of Dr. M. W. Townsend, deceased.

Dr. J. B. Murphy, of Chicago, is making a vacation tour in Mexico, where he is spending a few weeks in that balmy climate.

Dr. Adolph H. Urban, formerly of New York, has established his offices and residence at 523 Franklin Street, Buffalo, where he will devote himself to the practice of diseases relating to the ear, nose and throat. Hours: 9 to 12 a.m., 5 to 6 p.m., and by appointment. Telephone, Tupper 1436.

Dr. Thomas Lothrop, of Buffalo, a former editor of the Journal, has been suffering from grievous illness for a few weeks. Happily, however, just as these lines are printing, it is announced that he is very much improved and his early recovery is looked for.

Dr. Roswell Park, of Buffalo, was tendered a reception at Philadelphia, Wednesday evening, February 19, 1902, by the Charles K. Mills Neurological Society and the Pennsylvania Medical Society. On the evening referred to, Dr. Park presented a paper on Student life in the middle ages, before the societies in question. Upon invitation of the University Club in Buffalo, Dr. Park presented the same paper before that body on Saturday evening, March 15.

